# The Arduous Path Toward Equitable Access to Endocrinology Care

**DOI:** 10.1210/jendso/bvae134

**Published:** 2024-07-15

**Authors:** Giulio R Romeo, Tiziana Caputo, Izabela W Stanescu, Jamil B Alkhaddo

**Affiliations:** Joslin Diabetes Center, Harvard Medical School, Boston, MA 02215, USA; Beth Israel Deaconess Medical Center, Department of Medicine, Harvard Medical School, Boston, MA 02215, USA; Joslin Diabetes Center, Harvard Medical School, Boston, MA 02215, USA; Ambulatory Operation Services, Mass General Brigham, Harvard Medical School, Boston, MA 02215, USA; Division of Endocrinology, Department of Medicine, Allegheny Health Network, Pittsburgh, PA 15212, USA

**Keywords:** shortage of endocrinologists, health disparities, endocrinology access, care models, underrepresented in medicine

## Abstract

Multiple factors contribute to the widening gap between supply and demand of endocrinology services. In addition to the inadequate growth of the workforce, the inefficient utilization of endocrinologists’ expertise coupled with the rising prevalence of endocrine conditions has generated a crisis in access to specialty care. This mismatch is magnified in underserved communities and among certain racial/ethnic groups that carry a disproportionate burden of chronic diseases, like diabetes and osteoporosis, thus perpetuating the cycle of health disparities in vulnerable populations.

Reorienting the framework of endocrine care toward more effective and equitable access will require comprehensive changes in operational processes, system-based policies, and in the diversity of our workforce. Specifically, the progressive transition to outcome-driven, team-based models of care can extend endocrinology services beyond the traditional boundaries of in-office referrals and promote job satisfaction. Further, the implementation of policies that directly tackle structural determinants of health is a prerequisite to a more precise and equitable deployment of specialty care. In this view, the recruitment and professional growth of clinicians underrepresented in medicine along the career ladder, including leadership roles, is a key conduit to revitalize our field and to innovate the delivery of endocrine care across all communities.

The development and allocation of health-care resources is a fundamental nexus between medicine and society influenced by demographics, economic conditions, and policies and their guiding principles, among other factors. Inspired by social accountability, health-care systems aim to meet population needs in an equitable manner, prioritizing health concerns, and building on the collaboration of all stakeholders [1].

In this context, practicing physicians and professional associations have raised concerns regarding the deteriorating balance between supply and demand of physicians in multiple specialties and the heterogeneous availability of physicians’ expertise across communities. In 2022 the Association of American Medical Colleges released projections of a shortage of physicians ranging between 37 800 and 124 000 by 2034, both in primary and specialty care [2]. Specific to the endocrinology workforce, a significant mismatch between supply and demand is widening at a moment of unprecedented rise in the prevalence of and referrals for endocrine conditions, which has been associated with suboptimal delivery of endocrine services, especially in underserved communities [3].

Here we aim to discuss [1] the crisis of endocrinology care access [2], the implications of insufficient access to endocrinology care on health disparities, and [3] proposed interventions to promote endocrinology care delivery across all communities.

##  

### Crisis of Endocrinology Care Access

#### Issue of supply

The inadequate growth of the pool of adult endocrinologists, which had been accurately forecasted [4], results from the stagnant recruitment in fellowship programs [5] and a relatively high rate of retirement [4] that may have accelerated since 2019 (Fig. 1A). In part, these trends are fueled by low compensation, relatively to other subspecialties, and elevated risk of burnout, which negatively affect the perception of entering a career in endocrinology [5]. In addition, at least 20% of adult endocrinologists are primarily engaged in research, teaching, or administrative roles, thus further reducing the “stock” of patient-focused physicians. Conversely, some progress has been observed in regard to the diversity of the workforce, as women constitute 53% of the current pool of endocrinologists (https://www.aamc.org/data-reports/workforce/data/active-physicians-sex-specialty-2021) and prospectively 65% by 2030, owing to the highest percentage increase in female enrollees among fellowship training programs [6]. This sex shift has the potential to revitalize the perception of our field, especially if coupled with measures that narrow the gap of physicians underrepresented in medicine (URiM) and improve equity and inclusion in endocrinology (Fig. 1B) [7, 8].

**Figure 1.**
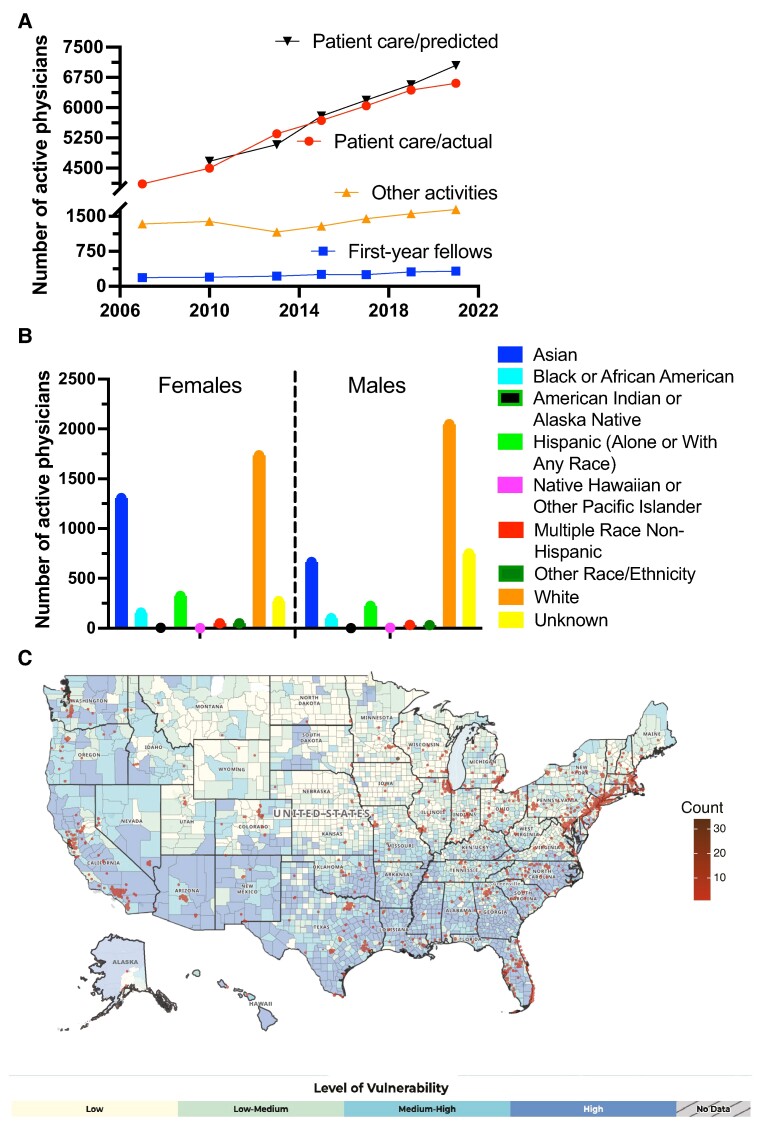
Trends in endocrinology workforce and distribution of adult endocrinologists in the United States. A, Trends of endocrinologists primarily active in patient care (red) or other activities (research, teaching or administrative roles; orange), and first-year endocrinology fellows (blue) between 2007 and 2021. The patient care/predicted plot (black) represents the anticipated number of patient-focused physicians based on the number of fellows graduating in the previous year, assuming no changes in attrition. Note gap between the actual and predicted pool of active physicians between 2019 and 2021. Data are extracted from the Association of American Medical Colleges https://www.aamc.org/data-reports/workforce/data/active-physicians-sex-specialty-2021 and National Residency Matching Program https://www.nrmp.org/match-data-analytics/fellowship-data-reports/. B, Breakdown of endocrinology workforce by self-identified race/ethnicity and sex in 2018. Data are extracted from the Association of American Medical Colleges https://www.aamc.org/data-reports/ workforce/data/table-12-practice-specialty-females-race/ethnicity-2018 and https://www.aamc.org/data-reports/workforce/data/table-13-practice-specialty-males-race/ethnicity-2018. C, Overlay of county distribution of adult endocrinologists by zip code and social vulnerability index (SVI). NPI data for endocrinologists was extracted from the National Plan and Provider Enumeration System (https://www.cms.gov/medicare/regulations-guidance/administrative-simplification/data-dissemination) and filtered by taxonomy code (207RE0101X). SVI map was extracted from https://www.atsdr.cdc.gov/placeandhealth/svi/data_documentation_download.html.

#### Issue of demand

On the other side of the scale, the increasing prevalence of common endocrine conditions [9], and growing disparities in their burden and outcomes among population groups [10, 11], pose the daunting task to meet the expected demand while tackling care inequities.

An analysis of the National Ambulatory Medical Care Surveys between 1999 and 2009 showed that the number of visits that would result in a referral to another physician had increased 159% nationally during that period [12]. Several factors contribute to the surge in referrals: increase in the complexity of management of endocrine disorders (eg, use of new medications for osteoporosis or advanced diabetes technology devices); higher frequency of incidental lesions that require endocrine evaluation; the overburden of primary care practitioners (PCPs) engendering delegation of larger aspects of care to specialists [13]; finally, lack of guidelines or predefined criteria for endocrinology referrals. While a definition of “appropriate” referral cannot be easily standardized across institutions, Mehrotra and colleagues [14] found that the rate of inappropriate referrals ranged from 0.7% to 65%.

#### Practice of longitudinal follow-ups

In the context of this rise in consultations, the modest workforce scalability and operational flexibility present the endocrinology community with a key question: Is it realistic to provide longitudinal care for a large group of established patients while maintaining meaningful access for new referrals? The Lewin Group reported that a full-time adult endocrinologist provides on average approximately 3000 visits yearly by working 38 to 40 hours weekly (exhibit 14c in https://www.endocrine.org/-/media/endosociety/files/advocacy-and-outreach/other-documents/2014–06-white-paper--endocrinology-workforce). However, patient panel size is estimated to range widely, between 1300 and 2000 per endocrinologist full-time equivalent, depending on the setting (eg, academic vs private practice), subspecialty focus, and the ratio of established patients to new consultations. Several factors perpetuate unnecessary longitudinal care in endocrinology clinics after the initial referral, despite evidence that many patients (eg, people with stable type 2 diabetes) can be effectively managed by their PCP team. First, the use of fee-for-service rather than value-based models does not incentivize seeing a higher proportion of new patients. In general, a specialist will more easily evaluate 2 established patients than 1 new referral within the same time interval, often collecting more relative valuable units. Second, the patient perceives that the support received by a specialist is essential to managing even a stable condition and invariably superior to the PC setting. As a corollary, clinicians are wary of creating patient dissatisfaction due to termination of a consultative rapport as it can result in unfavorable surveys. Third, the lack of periodic restratification of established patients (continuing specialty care vs returning to the referring team) fails to end the life cycle of a consultation. This inefficient workflow has generated endocrinology practices with burgeoning panels, but insufficient access for new referrals and generally long wait time.

### Both Geographical and Socioeconomic Barriers Contribute to Health Disparities in Endocrine Care

The insufficient access to endocrinology services affects all demographics, but its consequences are magnified in certain racial/ethnic groups that carry a disproportionate burden of endocrine conditions [3, 10, 15]. The prevalence of diabetes mellitus (DM) in US adults is significantly higher in racial and ethnic minority groups, when compared to non-Hispanic White (NHW) people, and is inversely correlated to family income and level of education [16]. In addition, the use of preventive practices, including eye exams and glycated hemoglobin A_1c_ (HbA_1c_) testing, is less common in people with lower education levels [17]. Further, escalation of treatment was less likely to occur for non-Hispanic Black (NHB) and Mexican American people, when goals of DM control were not achieved [18]. Finally, people with DM of race/ethnicity minority groups living in urban areas were more likely to have HbA_1c_ greater than 9%, when compared to NHW [19]. Similarly, certain ethnic groups may experience worse outcomes for other prevalent endocrine conditions [11, 20].

While addressing the complex underpinnings of social determinants of health extends beyond the scope of this article, the overlay of the distribution of adult endocrinologists in the United States with the map of social vulnerability index (https://www.atsdr.cdc.gov/placeandhealth/svi/data_documentation_download.html accessed December 1, 2023) reveals a mismatch in geographical access to endocrine care, especially in large rural areas of the South/Southwest and Midwest of the country (Fig. 1C). In addition to socioeconomic factors, lack of educational resources and distance to health facilities are important barriers to DM self-management for rural communities. Telehealth interventions can be effective in managing many endocrine conditions [21] but may be hampered by inadequate broadband connectivity or digital health literacy, which disproportionately apply to underserved communities (and minority race/ethnicity groups [22].

Irrespective of the geographic proximity to an endocrinology center, people from low socioeconomic status endure a lower likelihood of receiving endocrine care [23] or advanced treatment for osteoporosis after a fragility fracture [24], which reflects in part a substandard approach to referrals for specialized management. In addition, growing evidence indicates a disproportionately low access to DM technology devices [25] in certain race/ethnicity groups that results from the combination of deficient insurance coverage, inadequate pattern of prescriptions by health-care professionals, and limited provision of diabetes self-management education to support the use of these impactful tools. As a further example of structural racism, minority groups are less likely of being prescribed glucagon-like peptide–1R agonists [26] or PCSK9-inhbitors [27], when compared to NHW, despite a higher risk of cardiovascular morbidity and mortality.

Although operational changes are critical to expand the capacity for new patients, alone these efforts are not sufficient to eradicate disparities in access to endocrine care for vulnerable groups [28]. For instance, same-day cancellation or nonattendance to an endocrinology clinic was twice as high in people insured via Medicaid Managed Care, when compared with commercial carriers [29]. This information suggests that—even after securing an appointment—certain obstacles prevent appropriate access to specialty care, including costs related to travel, inadequate or high-deductible health insurance coverage, low health literacy, language barrier, and possibly lack of trust.

### Moving Past Business as Usual to Improve Equitable Access to Endocrine Care

Reorienting the framework of endocrine care toward more equitable access will require comprehensive changes in programmatic policies, operational processes, and in the diversity of our workforce. Through an iterative process, clinicians, policymakers, and patient advocacy groups are tasked with addressing structural drivers of health by devising novel models of care and reimbursement conducive to reduce health disparities for endocrine conditions.

#### Finding purpose in outcome-driven coordinated care

While increasing evidence indicates that coordination of care can improve outcomes, a cultural shift is the prerequisite to transform the fragmented PCP-patient-endocrinologist dynamic into a productive team-based collaboration. Physicians have been spending more time performing administrative tasks and less time on actual face-to-face interactions with patients. Coupled with the increasing pressure to meet relative valuable unit goals and other expectations, this situation has resulted in a rising rate of burnout for endocrinologists [30] (and virtually any other specialty). We believe that stratifying the life cycle of a consultation based on complexity can reduce unnecessary longitudinal care in endocrinology clinics and help control panel size and associated tasks that are often a major factor of burnout. The partnership with the patient and their referring clinician is essential at all stages, from the intake and triage process, to setting goals of the consultation, and optimizing the discharge plan, which should be accompanied by a clear communication of contingencies [31] and supported by other qualified resources in the PC setting (eg, pharmacist with expertise on diabetes management).

In addition, compensation models focused on outcomes could incentivize better integration of care, which is especially relevant for people in underserved communities who experience a higher burden of complex conditions [10, 15]. The progressive transition from fee-for-service to value-based care seeks to prioritize improvement of clinical (eg, reduction in HbA_1c_) and patient-centered benchmarks, and the coordination between specialists and PCPs, possibly with long-term cost reductions [32]. Multiple small-scale initiatives demonstrated the effectiveness of collaborative, performance-driven compensation plans [33], but they also exposed structural barriers for vulnerable groups of patients such as unpaid time off for visits and inadequate health-care literacy (eg, challenges with approval process of continuous glucose monitoring) [34]. Continued advocacy is needed to translate the experience of pilot programs into policy changes that affect population outcomes across all demographics. For instance, the recent proposal under the Medicare physician fee schedule to reimburse risk assessment for social determinants of health is an important step, as acknowledged by the Endocrine Society https://www.endocrine.org/advocacy/society-letters/2023/mpfs-2024, because it is linked to system-based interventions to mitigate identified determinants (eg, support of a care navigator).

#### Bringing endocrinology out of the endocrinology clinic

The inefficiency of the current system should encourage the deployment of endocrinology services beyond the traditional boundaries of in-office referrals. In this context, novel community-focused models of care should combat health inequities, tackle implicit bias [35], and promote cultural “concordance” between the health-care team and the patient [36]. Viable approaches include provision of support tools and education to PCPs, especially in rural areas (eg, Project-ECHO) [37], provider-to-provider electronic consults, and embedding endocrine specialists in PCP clinics, all of which can reduce the volume of lower-complexity referrals and enhance the quality of specialty care in community settings.

As endocrine care increasingly dovetails with numerous other conditions, endocrinology centers should champion a new vision for integrated management of chronic diseases. Multidisciplinary (eg, cardiometabolic) clinics could become a “hub” for innovation not only in care delivery but also in translational research, which is central to the intellectual legacy of endocrinology.

#### Amplifying diversity as a core value of the endocrinology workforce

Increasing recruitment and retention of URiM clinicians is essential for the future of endocrinology and the health-care ecosystem as a whole. Estimates from 2018 indicate that only 3.3% and 7.1% of US endocrinologists identify as Black/African American or Hispanic/Latinx, respectively (Fig. 1B). Beyond mission statements, embracing diversity and inclusion requires allocating resources for education and mentorship programs for URiM groups that offset the current gap in representation at all career levels, including the conspicuous imbalance in leadership positions [28]. Building on current efforts by medical organizations (eg, FLARE and ExCEL program), the endocrinology community should lead the way in nurturing medical students and residents from URiM groups, and foster their professional growth through the ranks as endocrinology faculty. The journey toward an equitable, antiracist workplace must include transparency of compensation practices and promotion criteria, closely monitored at all nodes of the career ladder. These measures should not come at the cost of disproportionate assignment of responsibilities with low likelihood of professional advancement, often referred to as the “minority tax” [38].

In addition to being an ethical imperative, addressing diversity in endocrinology is vital to job satisfaction. A survey administered by the AACE Diversity, Equity, and Inclusion committee indicated that approximately 52% of respondents (primarily endocrinologists) felt discriminated in the endocrine community, with sex, ethnicity, and country of origin as the most common reasons; 61% of respondents highly valued the importance of increasing diversity in endocrinology. Particularly relevant to the pool of endocrinologists in the United States, approximately 50% of women (vs 25% of men) felt they had to “work harder than their peers to be valued equally” [39].

In turn, changes in workforce representation could pave the way to novel models of patient-centered, culturally-sensitive care, especially in underserved communities. Of note, standards for culturally and linguistically appropriate services to reduce health-care disparities rely on increasing the diversity of the clinical team and cultural “concordance” [40].

In summary, the endocrinology supply-demand mismatch should inspire new approaches of care delivery that maximize the utilization and effect of endocrinology expertise, and facilitate equitable access across all communities. The professional development of a diverse group of leaders is a key conduit to this vision and can have a ripple effect in all domains of academic medicine while revitalizing the appeal of entering a career in endocrinology.

## Data Availability

Some or all data sets generated during and/or analyzed during the current study are not publicly available but are available from the corresponding author on reasonable request.

## Abbreviations

DM: diabetes mellitus
HbA_1c_: glycated hemoglobin A_1c_
NHB: non-Hispanic Black
NHW: non-Hispanic White
PCP: primary care practitioner
URiM: underrepresented in medicine
